# Supplementary figures and images for: Comparison of metabolic states using genome-scale metabolic models
Source: PLoS Comput Biol. 2021 Nov 8;17(11):e1009522. doi: 10.1371/journal.pcbi.1009522 (PMC8601616; doi:10.1371/journal.pcbi.1009522)

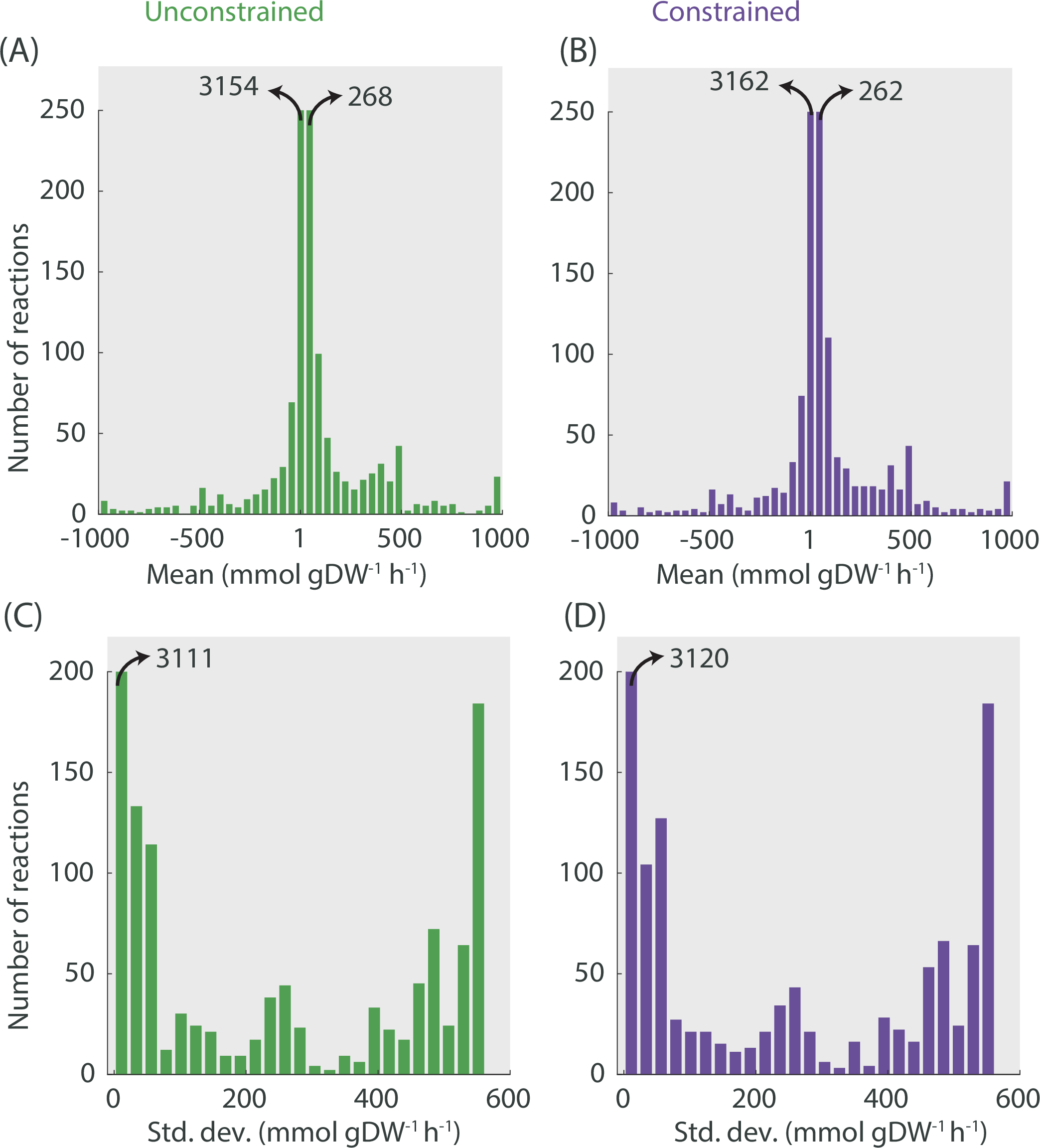

Supplement: S1 Fig — (A) Histogram of reaction flux means from the unconstrained and (B) constrained simulations. (C) Histogram of reaction flux standard deviations from the unconstrained and (D) constrained simulations. Due to the very high number of reactions for the lowest values, their number of reactions have been indicated separately with arrows. (TIF) [file pcbi.1009522.s001.tif]

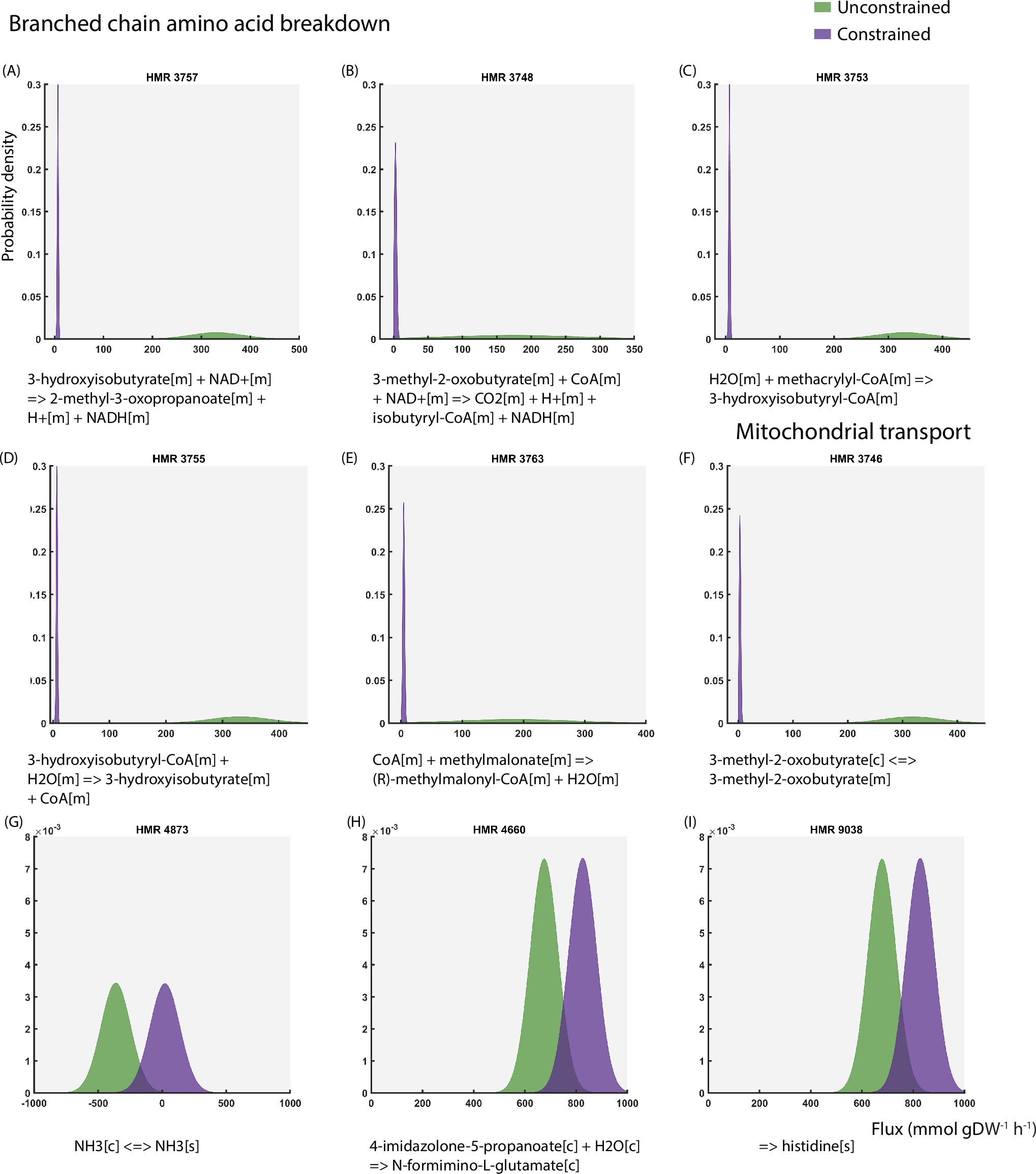

Supplement: S2 Fig — The reaction IDs and chemical equations have been shown above and below the plots respectively. (TIF) [file pcbi.1009522.s002.tif]

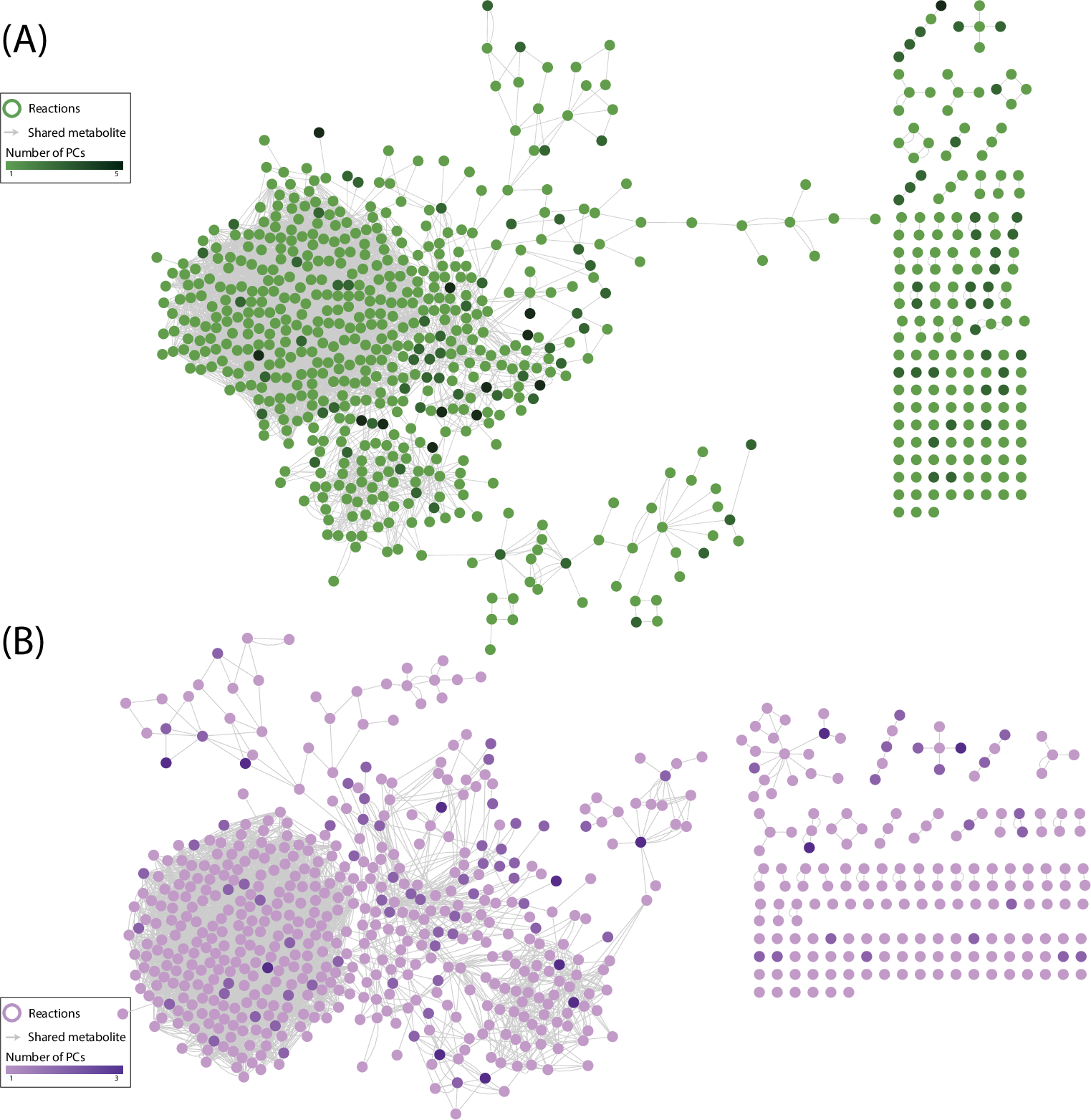

Supplement: S3 Fig — The two networks represent the global modules from (A) unconstrained and (B) constrained adipocyte network. Nodes represent reactions and the edges indicate shared reactant/product. Node colour is mapped to the number of involved modules. The NDEx links https://bit.ly/globalModulesUncon and https://bit.ly/globalModulesCon can be used to study the networks interactively. (TIF) [file pcbi.1009522.s003.tif]

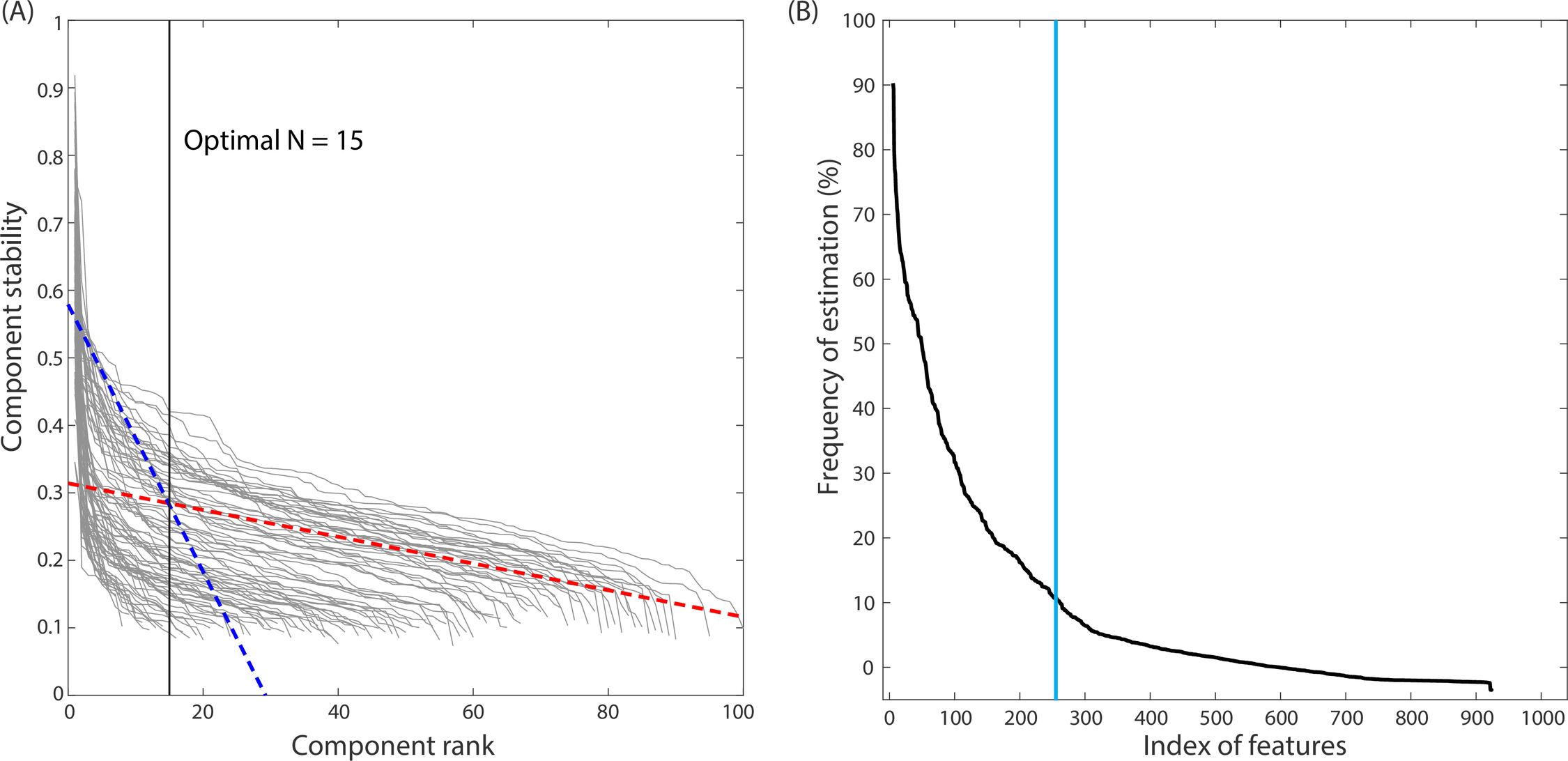

Supplement: S4 Fig — (A) Grey lines show the stability profiles of the bootstrapped Independent Component Analysis (ICA) runs. Blue and red dashed lines are results of two-line clustering with the optimal N determined as the point of their intersection (black vertical line). (B) Frequency of estimation of a feature as an independent component. Cyan vertical line marks the knee-point cut-off (288) for selecting distinct features. (TIF) [file pcbi.1009522.s004.tif]

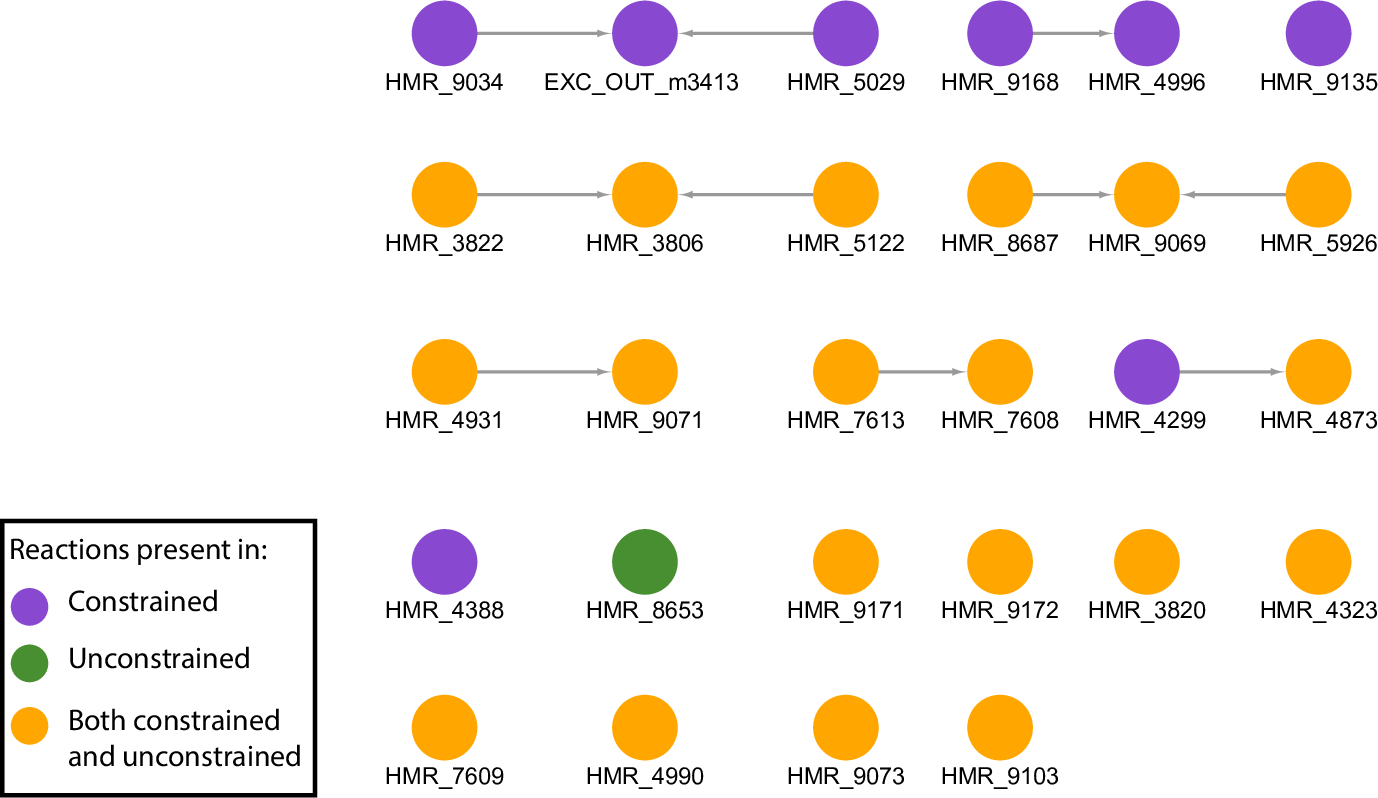

Supplement: S5 Fig — (TIF) [file pcbi.1009522.s005.tif]
