# Supplementary material for: Comparison of metabolic states using genome-scale metabolic models
Source: PLoS Comput Biol. 2021 Nov 8;17(11):e1009522. doi: 10.1371/journal.pcbi.1009522 (PMC8601616; doi:10.1371/journal.pcbi.1009522)
Supplement: Attachment — Submitted filename: Report_ComMet.pdf [file pcbi.1009522.s012.pdf]

Report

Comparison of metabolic states using genome-scale metabolic models

The authors present a method, called ComMet, which is able to combine two cutting edge tools for analyzing metabolic networks: the metabolic reconstruction provided in Braunstein et al. and the Principal Component Analysis applied to flux-flux covariance matrix as in Barret et al. They apply ComMet to two genome-scale metabolic networks differing in the presence or absence of the branched-chain amino acids (BCAAs), considered as reliable biomarkers for obesity. Overall, the pipeline presented by the authors is solid and the results are convincingly validated by comparing ComMet predictions to known results. Moreover, the method brings to light new compensatory mechanisms that are present in absence of BCAAs.

This approach benefits from the efficiency of the metabolic reconstruction provided in Braunstein et al. and the informative decomposition scheme described by Barret et al. I find the manuscript well written and of high impact in the field of metabolic fluxes. However, I have some major remarks to address.

Major points

- I find the explanation of the method presented in lines 54-56 inconsistent with the name used within the manuscript, that is Sampling. It seems that the method in Braunstein et al. produces an approximation of the marginal probability of the fluxes, avoiding the sampling of the solution space (as it is well described in lines 122-148). I kindly suggest renaming the method as it may be misleading. A similar remark deals with the presentation of the Principal Component Analysis method in lines 60-61. The authors of Barret et al. have indeed obtained a covariance matrix from sampled configurations of fluxes, but probably the authors may anticipate that they will apply the PCA analysis directly to the covariance matrix provided by Braunstein et al., without the use of sampled configurations.
- Lines 95-96. One of the two conditions analyzed by the authors assumes that the uptake of BCAAs is unlimited. However, after the pre-processing (Flux Variability Analysis) in lines 432-433, the corresponding reactions may be limited by stoichiometric constraints. What is the final range of variability for these reactions, after the pre-processing? I believe that this information may underline how different is the unconstrained scenario from the constrained one.
- I have noticed that in Fig. S1 a large fraction of the fluxes has an expected value close to the upper and lower bound of the flux variability, suggesting that these results strongly depend on the arbitrary value of the lower and upper bounds (-1000, 1000). This may reveal the presence of unfeasible thermodynamic cycles in the genome-scale metabolic network, which allow the fluxes to take, in absolute value, "infinite" values (see 10.3390/metabo3040946). I suggest checking for this possible issue.

- The PCA is entirely developed as described in Barret et al. However, the authors may perform a simpler analysis by projecting, for instance, the covariance matrix of the constrained data to the principal components obtained from the unconstrained case. How different is the reconstructed covariance matrix from the "true" one? The differences may already give some biological interpretation of the two studied scenarios. Finally, have the authors performed a clustering analysis of the covariance matrices? Are there some clusters that are in agreement with the network analysis proposed in Fig. S3?
