# Supplementary material for: Comparison of metabolic states using genome-scale metabolic models
Source: PLoS Comput Biol. 2021 Nov 8;17(11):e1009522. doi: 10.1371/journal.pcbi.1009522 (PMC8601616; doi:10.1371/journal.pcbi.1009522)
Supplement: Attachment — Submitted filename: ComMet_responses_plosCB.pdf [file pcbi.1009522.s013.pdf]

Response to reviewers

Editorial comment

While the reviewers appreciated the attention to the research topic, they raised several substantial concerns about the manuscript based on which it cannot be accepted in its present form. In particular, reviewer #2 expressed concerns about the methodological and conceptual advance the study provides. However, we are willing to consider a substantially revised version in which all major issues raised (especially by reviewer #2) have been adequately addressed. It would be crucial i) to demonstrate that the predictions made by the ComMet workflow are different from what can be obtained by simpler approaches and ii) to provide some further validation of the predictions based on literature data. Please make sure that your revision addresses the specific points made by each reviewer.

We thank you for the feedback and remarks. We have revised the manuscript to address all the points raised by the reviewers. Please find our detailed responses to specific points made by the reviewers below, along with references to the corresponding lines in the revised manuscript. Through our answers, we hope that we have addressed all the concerns raised by the reviewers and demonstrated the novelty and conceptual advancement of our study in a convincing manner.

Reply to Editor's points:

As the Editor's point (i) is similar to the last question of Reviewer 2, we have responded to this point under the last question of Reviewer 2 below.

*Regarding Editor's point (ii), we have added more literature references (Snell and Duff (1977), Tischler et al., (1980) and Salinas-Rubio et al., (2018)) and derived additional interesting interpretations of ComMet results while studying some independent components as a whole (lines **334-344 and 435-444**). We have now also included this under the Results section of the manuscript.*

Kindly note that all the line numbers mentioned in this response letter correspond to the line numbers in the revised manuscript and not to the version with tracked changes.

Reviewer 1

Report

Comparison of metabolic states using genome-scale metabolic models The authors present a method, called ComMet, which is able to combine two cutting edge tools for analyzing metabolic networks: the metabolic reconstruction provided in Braunstein et al. and the Principal Component Analysis applied to flux-flux covariance matrix as in Barret et al. They apply ComMet to two genome-scale metabolic networks differing in the presence or absence of the branched-chain

amino acids (BCAAs), considered as reliable biomarkers for obesity. Overall, the pipeline presented by the authors is solid and the results are convincingly validated by comparing ComMet predictions to known results. Moreover, the method brings to light new compensatory mechanisms that are present in absence of BCAAs. This approach benefits from the efficiency of the metabolic reconstruction provided in Braunstein et al. and the informative decomposition scheme described by Barret et al. I find the manuscript well written and of high impact in the field of metabolic fluxes. However, I have some major remarks to address.

Major points

- I find the explanation of the method presented in lines 54-56 inconsistent with the name used within the manuscript, that is Sampling. It seems that the method in Braunstein et al. produces an approximation of the marginal probability of the fluxes, avoiding the sampling of the solution space (as it is well described in lines 122-148). I kindly suggest renaming the method as it may be misleading. A similar remark deals with the presentation of the Principal Component Analysis method in lines 60-61. The authors of Barret et al. have indeed obtained a covariance matrix from sampled configurations of fluxes, but probably the authors may anticipate that they will apply the PCA analysis directly to the covariance matrix provided by Braunstein et al., without the use of sampled configurations.

  Reply: *We thank the reviewer for noticing this. As the authors of the Braunstein method (Braunstein et al., 2017) did not introduce a concise expression/term to describe their approach, we used the term "sampling", however, as per the reviewer's suggestion, we now refer to it as "analytical approximation of fluxes" to avoid any ambiguity, and we explicitly define this term in the manuscript in lines* ***63-64****.*
  As for the Principal Component Analysis (PCA), we indeed apply the PCA decomposition on the covariance matrix resulting from the Braunstein et al. approach and we have clarified this in lines ***116-117****.*

- Lines 95-96. One of the two conditions analyzed by the authors assumes that the uptake of BCAAs is unlimited. However, after the pre-processing (Flux Variability Analysis) in lines 432-433, the corresponding reactions may be limited by stoichiometric constraints. What is the final range of variability for these reactions, after the pre-processing? I believe that this information may underline how different is the unconstrained scenario from the constrained one.

  Reply: *We thank the reviewer for their observation and their comment. After the preprocessing, the final range of variability of BCAA uptake reactions is still 0-1000 mmol/gDW/h in the unconstrained case, while both upper and lower bounds are 0 mmol/gDW/h in the constrained case.*

  On this occasion, we would like to clarify that, in the constrained condition, we had previously applied the preprocessing before the application of the BCAA uptake constraints. We have now ensured that the preprocessing steps are applied after the application of any constraints, and re-ran the rest of ComMet's workflow on both

constrained and unconstrained cases. Although this did not lead to any appreciable differences in the resulting modules, there are a few minor changes in the numbers reported previously (for example, the number of principal components describing 99.9% variation go from 514 to 515 in the constrained simulation). Please note they did not affect the conclusions we had presented in the earlier version of this manuscript.
We have now revised the Results section and updated all the figures 1-7 and S1-S5 in the manuscript. Kindly refer to the revised version of the manuscript with tracked changes for all the aforementioned updates.

- I have noticed that in Fig. S1 a large fraction of the fluxes has an expected value close to the upper and lower bound of the flux variability, suggesting that these results strongly depend on the arbitrary value of the lower and upper bounds (-1000, 1000). This may reveal the presence of unfeasible thermodynamic cycles in the genome-scale metabolic network, which allow the fluxes to take, in absolute value, "infinite" values (see 10.3390/metabo3040946). I suggest checking for this possible issue.

  Reply: *We thank the reviewer for their suggestion. In order to check the thermodynamic feasibility, we applied the COBRA function checkThermodynamicConsistency on the preprocessed models from both the cases. We set the objective function in both cases to the reaction forming lipid droplet (HMR_obj) and used the gurobi solver. This analysis did not reveal any unfeasible thermodynamic cycles.*
  We have now included this additional suggested check by the reviewer in lines ***603-608*** *of the manuscript as well for more clarity.*

- The PCA is entirely developed as described in Barret et al. However, the authors may perform a simpler analysis by projecting, for instance, the covariance matrix of the constrained data to the principal components obtained from the unconstrained case. How different is the reconstructed covariance matrix from the true one? The differences may already give some biological interpretation of the two studied scenarios. Finally, have the authors performed a clustering analysis of the covariance matrices? Are there some clusters that are in agreement with the network analysis proposed in Fig. S3?

  Reply: *We thank the reviewer for giving us an opportunity to clarify. Our objective was to characterise the differences between the flux spaces from the constrained and unconstrained cases. ICA was the method of our choice to identify these distinct and condition-specific biochemical modules. Clustering covariance matrices directly (without PCA) would miss the components/biochemical modules of individual flux spaces and thus, would yield a less clear view of the biological differences.*

  Prior to selecting ICA as the approach for comparing the conditions, we had applied a clustering analysis on the principal components (using Hierarchical Clustering based on pairwise Euclidean distances) to identify components that were similar and different between the conditions. However, this analysis did not yield distinct clusters as expected

and thus, was unable to extract meaningful differences between the conditions being compared.

We also note that Fig. S3 depicts a network of global modules for a single condition and contains the reaction modules extracted from all the principal components from that condition. These modules could not be compared to any clusters as no distinct clusters could be identified from the aforementioned analysis. On the other hand, Fig. 5 shows the combined network of reaction modules extracted from the principal components that were identified by ICA as distinct between the conditions.

Following the reviewer's comment, we have mapped the reactions from Fig. 5 on the corresponding network in Fig. S3 as it may not have been clear previously. This mapping can be seen in Fig. R1 below, where, Fig. R1 (A) shows the distinct unconstrained modules from Fig. 5 mapped on the global unconstrained network (Fig. S1 (A)) and Fig. R1 (B) shows the distinct constrained modules from Fig. 5 mapped on the global constrained network (Fig. S1 (B)). Fig. R1 shows that all the reactions from distinct modules mapped to the respective global network and this overlap implies that the distinct modules are indeed a subset of the global network. The modules distinct between the conditions are highlighted prominently in Fig. 5. We hope that this figure and the above explanation addresses the reviewer's questions. We would be happy to provide additional clarifications if the reviewer deems necessary.

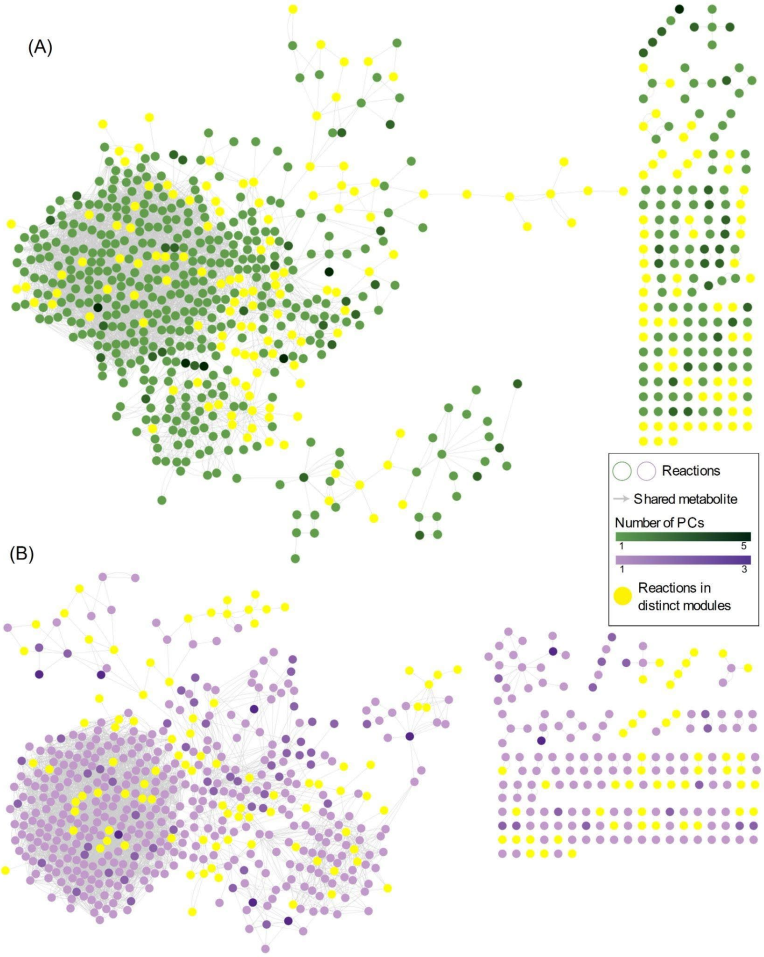

Fig R1: Overlap between the reactions in distinct modules (Fig. 5) and global modules (Fig. S3) in (A) unconstrained and (B) constrained cases.

Reviewer 2

Authors extend the previous work done in this field by the Palsson group for the analysis of sampled flux distributions using PCA only that in this study they use distributions obtained from analytic approximation published recently. They also add an ICA piece to extract some additional modules that is marginally and incrementally different from the previous work in this area. They then show the workflow for the analysis of metabolism under two different cases, first when there are no constraints and then second, when there are constraints on the uptake of specific AA (Leucine, Valine and Isoleucine). The work seems technically interesting but I am not sure this work has enough conceptual or methodological advance for PLoS CB and neither are the insights identified in this study.

Reply: *We thank the reviewer for their summary and comments. We have addressed their comments in detail below and we hope that we have clarified the novelty in a convincing manner. We have also revised our manuscript which now emphasises the conceptual advancement of our study (lines* ***479-510*** *) and we also explain in the second comment of Reviewer 2 below.*

Major Concerns

The paper is not structured well. It is written reasonably well but for example there are description of the methods in the results section (Lines 95-Line 120).

Reply: *We thank the reviewer for their feedback. As per their suggestion, we have moved the technical details into the Methods section. We would like to note that we have followed the Intro-Results-Discussion-Methods structure according to the guidelines of PLoS Computational Biology and therefore mentioning some methods in the results section is inevitable. To understand the detailed results, the readers must have an overview of our method, but we now only provide a high-level summary of ComMet in the Results section (lines **95-134**).*

The major weakness is that the paper does not identify what specific aspects of the workflow is novel compared to Barrett paper that also applied PCA to the actual sampled distributions as opposed to the analytic distribution. A proper comparison would be to look at the differences and perhaps show that the PCA of the analytic piece is different and produces some new insights that was not obvious with the previous method of PCA on the sampled distributions as opposed to the approximate analytical distributions. If the advance is computational efficiency then authors should provide data supporting these claims. This reviewer seriously wonders if sampling based on ACHR is indeed a limitation at all.

Reply: *We thank the reviewer for giving us an opportunity to clarify. We understand the reviewer's perspective that the novelty of ComMet may not have been conveyed clearly. In the following paragraphs, we explain (i) differences between the Barrett study (Barrett et al., 2009) and our study, (ii) the innovations of our study and (iii) the rationale for using the analytical approximation scheme (by Braunstein et al., 2017).*

(i) Differences between the Barrett study and our study:

There are two main differences between the Barrett study and our study which we elaborate below using a schematic. Fig. R2 compares the ComMet workflow with that presented in the Barrett study.

First and foremost, the Barrett study only uses a principal component-based decomposition to analyse the structure of a single flux space. Whereas, we use such decompositions to compare and extract the distinct metabolic differences between multiple flux spaces. The decomposition of an individual flux space (Fig. R2, dotted box) provides a basis for how to analyse and subsequently compare flux spaces. Prior to identifying distinct features between the metabolic states simulated in our study (Fig. R2, red box), we used the PCA based decomposition from the Barrett study to extract the characteristic biochemical features from each flux space (Fig. R2, dotted box). This decomposition is the only aspect common between the Barrett study and ComMet (Fig. R2, dotted box).

Secondly, the model size targeted by both studies are different. Our approach is specifically aimed at large-scale GEMs like human models and human tissue models, whereas, a much smaller E. coli model was used in the Barrett study. Due to their sheer size and complexity, human GEMs are challenging to analyse and interpret. In addition, selecting parameters for existing analysis methods such as metabolic objective functions and model constraints is not straightforward in human GEMs. In our study, we developed a method that facilitates analysis of

large flux spaces independent of objective and media specifications which was not addressed in the Barrett study.

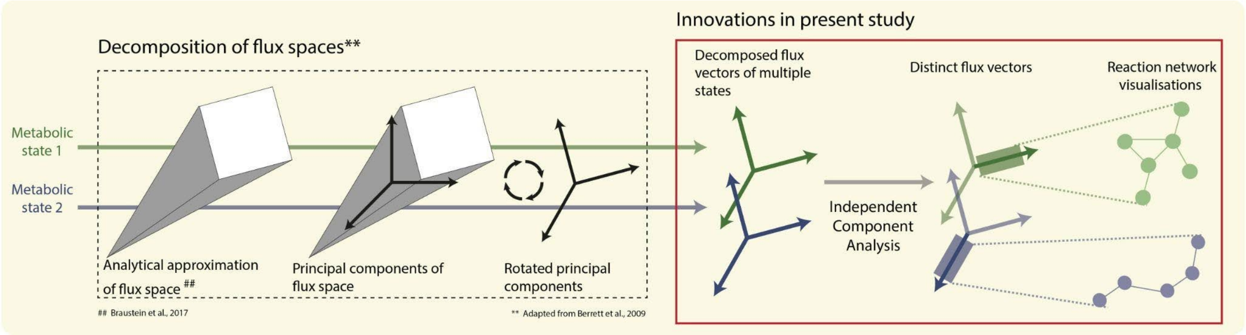

Fig. R2: Schematic of the workflow underlying ComMet that shows the steps that are different between the Barrett study (dotted box) and our study. The steps within the red box highlight the innovations of the present study.

<u>(ii) Innovations of our study:</u>

In summary, the central conceptual advance of ComMet lies in its ability to compare multiple flux spaces, extract distinct metabolic features and visualise the extracted features (Fig. R2, red box).

We use the principal component based approach to decompose the flux space of the adipocyte model in two different metabolic states and show that modules similar to those presented by Barrett can also be obtained in large-scale models (Fig. S5). Following this decomposition, we extended the analysis of flux spaces by introducing the central aspect of comparing decomposed flux spaces, which was not trivial at all. As already described above under question 4 of Reviewer 1, we applied different clustering approaches before selecting ICA. To ensure that ICA indeed extracts distinct components, we carefully optimized several parameters needed to run ICA (bootstrapping to identify the optimum number of independent components, iterations to ensure reproducibility and frequency of feature estimation).

Moreover, we also present three novel, reusable visualisation strategies which facilitate comprehensive understanding of the network behaviour under the imposed constraints. These strategies look at the modules from three different angles, with each angle offering a unique perspective to interpreting the modules. The reaction map/network in Figures 5 combines the reactions from all the distinct modules into one network and showcases the reaction connectivity across the network. Additionally, grouping of these reactions based on subsystems provides an overview of the metabolic processes affected by our constraint introduction. The metabolic map shown in Fig. 6 zooms into subsystems (selected from Fig. 5) and reveals the underlying reactants and products. This representation offers a conventional pathway-like view and provides an indication of how the flow of metabolites in a subsystem may be affected by our constraint introduction. Fig. R3 (also Fig. 8) focuses on a single module identified to be distinct between the two conditions in Fig 5. This view shows reaction connectivity within a module and indicates which reactions show correlated behaviour within that principal component. This allows detailed examination of the reaction sets that govern specific biochemical aspects of the underlying metabolic state.

*Taken together, the innovative aspect of comparing large flux spaces and visualisation strategies that we introduce enables investigating a wide range of metabolic conditions even in human-sized GEMs which are highly relevant in a biomedical context. As the novelty of ComMet may not have been clear previously, we have now emphasised it under a new subsection "Innovations of the present study" the Discussion (lines **479-510**).*

<u>(iii) Reasons for using analytical approximation scheme:</u>
*Previous studies have shown that obtaining sampled distributions is very time-consuming (Schellenberger and Palsson (2009), Megchelenbrink et al., (2014) and Braunstein et al., (2017)). As our focus was on analysing flux spaces of large genome-scale metabolic models, we needed a computationally efficient method for obtaining probability distributions of reaction fluxes. In their paper, Braunstein et al. already demonstrated a tremendous computational advantage of their analytical approximation scheme over ACHR sampling even for models with thousands of reactions. For example, estimates of probability distributions were obtained from Recon1 (with x reactions) in 1 hour with their approach vs 20 days with ACHR in their tests (Fig. 2c in Braunstein et al.). Thus, we used the analytical flux space approximation solely to address the technical challenges arising from our large system of interest (Fig. R2, dotted box). Our study benefited from the computational efficiency of the Braunstein method and enabled obtaining a flux space probability distribution and covariance matrix for a human-sized GEM (Fig. R2, dotted box). Nevertheless, we would like to point out that the ComMet workflow can be applied to smaller models. In that scenario, the ComMet workflow may be adapted to obtain probability distributions of reaction fluxes either using the analytical approximation scheme or with Monte Carlo sampling as that would be computationally feasible (lines **513-518**). We have reiterated the rationale behind choosing the analytical approximation in lines **56-62 and 547-553** of the revised manuscript.*

The modules identified globally for the adipocyte network is then analysed but it is not clear whether the reactions constrained truly represent the physiology under obese/nonobese conditions before we can conclude that the changes in the flux space due to the lack of these constraints are meaningful. Furthermore the fact that one of the conditions is unconstrained space is not justified clearly. If the aim of the paper is to demonstrate a workflow then it should be illustrated on a small toy network so that it is clear what the advantages of the workflow are. If the goal is to understand metabolism of adipocytes under disease conditions then more physiology constraints and arguments are needed with some validation based on data from literature.

***Reply:** We thank the reviewer for their observation and feedback. We would like to point out that the main objective of this study was to demonstrate how factors underlying the differences between metabolic conditions can be identified for large-scale networks without resorting to prior assumptions like objective functions. Our aim was to showcase the new workflow on a large network and we used the iAdipocyte1809 model as "the toy network". We note that "toy networks" are usually simple systems and serve illustrative purposes. The adipocyte model may not be a small toy network but it serves the dual purpose of demonstrating the workflow and its potential biological relevance (lines **524-531**). The presence/absence of BCAAs for an adipocyte network serves as a proof-of-principle for how the method can identify relevant metabolic differences between conditions of interest (lines **99-100**) but is not meant to "truly represent the physiology*

under obese/nonobese conditions". The unconstrained condition serves as an ideal scenario where BCAAs and other nutrients are available for adipocytes. With a perturbation of merely 3 reactions, our approach identified several network-wide downstream effects. Modelling adipocyte metabolism in obese/non-obese conditions is beyond the scope of the current work. Nevertheless, as mentioned in lines ***532-535****, when suitable experimental data (such as gene expression) is available, specific disease conditions can be modelled and used in place of the simulated conditions in this study. We explain the reason for using the adipocyte model and the relevance of the simulated scenario in lines* ***524-529*** *of the revised manuscript.*

Authors apply ICA to the filtered PCA results and the value of this step is not at all clear. First of can the authors compare these results with what happens when ICA is done on the entire space compared to filtered results. Why do we need the PCA step ?

Reply: *We thank the reviewer for the opportunity to clarify. In their study, Barrett et al demonstrated the use of PCA on sampled flux space to obtain biochemically interpretable decomposition of the steady flux states. Using their approach, they decomposed the steady flux states of an E. coli model (123-dimensional space) into merely twenty distinct sets of reactions. Furthermore, they showed that these reaction sets operated independently to determine the flux state of the entire network under a given growth environment. Our objective was to analyse and compare metabolic states in large GEMs. However, selecting parameters for existing analysis methods such as metabolic objective functions and model constraints is not straightforward in human GEMs. Therefore, we sought to establish a method based on feasible flux space analysis as these approaches are independent of the above mentioned assumptions. In our approach, the principal component decomposition of an individual flux space was useful in obtaining biochemically interpretable reaction sets of the adipocyte model (in both conditions). Thus, this decomposition provided a basis for the subsequent comparison of flux spaces. We explain the rationale behind the PCA step in lines* ***64-74 and 539-546*** *of the revised manuscript.*

Regarding the filtering of principal components: As Fig. 4 shows, nearly the entire flux space can be recovered through ~500 principal components out of ~4000 in a given flux space. As the principal components that we remove prior to comparison explain only about 0.1% of variation of their flux space, they would not yield much information on the differences between flux spaces either. Retaining the components that are not significant determinants of metabolic behaviour could introduce noise in the subsequent analysis. We would also like to note that ICA is the most time consuming step of the ComMet workflow (Table S6). If the remaining components were included for ICA based comparison, it would also increase the columns in the input matrix for ICA (from ~1000 to ~8000) and thus invariably resulting in a tremendous increase in runtime. As the rationale behind filtering of principal components may not have been clear previously, we have now emphasised it in lines ***673-682****.*

We hope that our explanation answers the reviewer's concern and we would be happy to clarify any follow up questions the reviewer may have.

Also the reaction modules identified by ICA, how are they different from the subsystems annotation. Perhaps authors can compare these and show some additional modules.

Reply: *We thank the reviewer for their comment and for the opportunity to clarify. Subsystems on their own provide merely one form of annotating reactions in genome-scale models. They can be considered equivalent to pathways and are classified based on the type of biological macromolecule (for example Protein, fat, carbohydrate). As subsystems represent the biochemical processes in a cell, the subsystem annotations in GEMs remain fairly same across organisms and cell types. On the other hand, the reaction modules identified in this study (Fig. 5) specifically describe the most significant functional differences between the conditions studied. Reactions from a module are extracted from one component and can either belong to one subsystem or span across several subsystems. We identified two such examples from the network in Fig. 5 and have shown in Fig. R3 below. Fig R3 (A) shows the module from component 489 (unconstrained model) which contains reactions from the subsystems TCA Cycle, BCAA metabolism, Mitochondrial Transport, Arginine and proline metabolism and Leukotriene metabolism. On the other hand (B) shows the reactions extracted from component 354 (constrained model) where all the reactions belong to the subsystem Amino sugar and nucleotide sugar metabolism. Please note that only 4 out of 28 reactions in this*

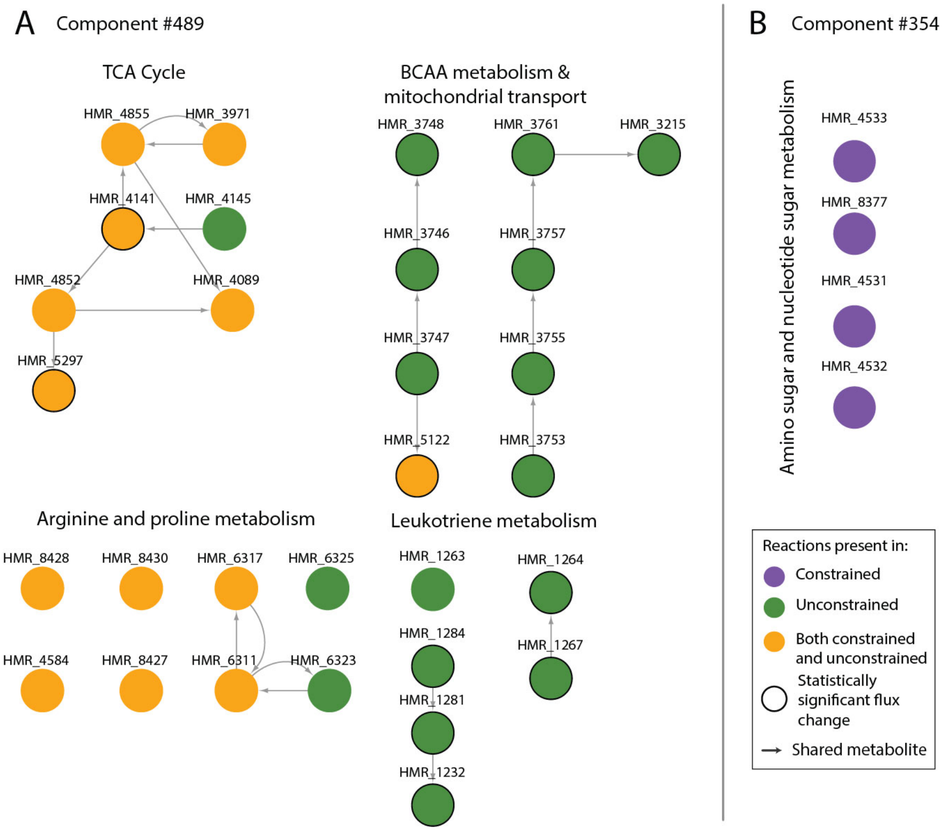

Fig. R3. Visualisation of the reaction sets in individual modules from components (A) #489 from the unconstrained model and (B) #354 from the constrained model.

We would like to use this opportunity to clarify that we combined the reactions from all the distinct modules to construct the reaction map/network shown in Fig. 5. We grouped the reactions based on subsystem annotation provided in the model to indicate which metabolic processes are

affected by the constraint introduction. Additionally, we laid out functionally similar groups together to highlight the process-level crosstalk across the metabolic network. Please note that the individual groups seen in Fig. 5 prominently feature the reactions from that corresponding subsystem. Additional details about the distinct module reactions shown in Fig. 5 are provided in Tab S5 and this network can be studied interactively using the NDEx link - (https://bit.ly/DistinctModules). Taken together, we hope that we have illustrated that modules not only describe metabolic functionalities across subsystems but are also biologically meaningful to our research question. As this may not have been clear previously, we have explained it in the new subsection, "Distinct modules and Subsystem annotations" under Results (lines ***425-444)*** *in the revised manuscript and included Fig. R3 as Fig.* ***8****.*

May be even compare with gene expression data as well to incorporate some of the work that the Palsson group has done around imodulons.

Reply: *We thank the reviewer for their interesting suggestion. As mentioned above and in lines* ***532-535*** *of the manuscript, ComMet can definitely use gene expression data in place of simulated states to constrain GEMs to specific conditions. This is being pursued in a follow-up study. Although comparing the ComMet modules to iModulons is beyond the scope of the current work, it is a very interesting direction to pursue in a future study.*

Finally the so called predictions of the ComMet workflow seems a bit trivial and easily predictable from a pure FBA simulation. Can the authors indicate why one would need to go through this entire exercise if we can use FBA or other sampling/coupling methods itself ?

Reply: *We thank the reviewer for their comment and the opportunity to clarify. ComMet and FBA are fundamentally different approaches that address two different research questions and have different technical requirements. Setting a metabolic objective and defining precise quantities of media metabolites are indispensable for a meaningful FBA simulation. For human GEMs, selecting an objective function is not as straightforward as biomass production and requires careful consideration of the underlying physiology. Contrastingly, the primary goal of ComMet is to enable a feasible analysis of large flux spaces independent of objective and media specifications. Therefore, it is not reasonable to compare ComMet with FBA.*

Nevertheless, as per the reviewer's suggestion, we performed FBA simulations on the same models used in our study. To choose the objective function, we looked into the physiological role of adipocytes. Adipocytes store energy in the form of lipid droplets whose size varies with changes in physiology. While developing the iAdipocytes1809 model, Mardinoglu et al., (2013) evaluated the function of adipocytes by estimating the formation of lipid droplet formation. Therefore, for our analysis, we also assumed lipid droplet formation as the objective function. FBA was run on both constrained and unconstrained models to maximise lipid droplet formation. FBA predicted 398 reactions with differing fluxes between the two models. We then compared these reactions to those that ComMet identified as distinct (i.e., reactions in Fig. 5 and Tab S5) and found 104 reactions to be common between FBA and ComMet predictions. The reactions that don't overlap could very well be due to the assumptions made for running FBA.

Due to the methodological differences in ComMet and FBA, they are useful in very different scenarios. We would like to emphasise that ComMet is an approach that is not intended to compete with FBA and complements the existing methods for conducting studies with GEMs. ComMet is recommended for cellular systems, particularly in human tissue models, where assuming an objective is tricky and when accurate metabolite constraints are unavailable or difficult to obtain. In our revised manuscript, we clarify the differences between FBA and ComMet in lines ***554-565*** *and provide examples of potential applications of ComMet in lines* ***523-535****.*

References

1. *Braunstein A, Muntoni AP, Pagnani A. An analytic approximation of the feasible space of metabolic networks. Nature Communications. 2017;8(1). doi:10.1038/ncomms14915.*
2. *Barrett CL, Herrgard MJ, Palsson BO. Decomposing complex reaction networks using random sampling, principal component analysis, and basis rotation. BMC Systems Biology. 2009;3(1):30. doi:10.1186/1752-0509-3-30.*
3. *Mardinoglu A, Agren R, Kampf C, Asplund A, Nookaew I, Jacobson P, et al. Integration of clinical data with a genome-scale metabolic model of the human adipocyte. Molecular Systems Biology. 2013;9(1):649. doi:10.1038/msb.2013.5.*
4. *Megchelenbrink, Wout, Martijn Huynen, and Elena Marchiori. "optGpSampler: an improved tool for uniformly sampling the solution-space of genome-scale metabolic networks." PloS one 9.2 (2014): e86587. doi:10.1371/journal.pone.0086587*
5. *Salinas-Rubio D, Tovar AR, Torre-Villalvazo I, Granados-Portillo O, Torres N, Pedraza-Chaverri J, et al. Interaction between leucine and palmitate catabolism in 3T3-L1 adipocytes and primary adipocytes from control and obese rats. The Journal of Nutritional Biochemistry. 2018;59:29-36. doi:10.1016/j.jnutbio.2018.05.011.*
6. *Schellenberger, J., & Palsson, B. Ø. (2009). Use of randomized sampling for analysis of metabolic networks. Journal of biological chemistry, 284(9), 5457-5461. doi: 10.1074/jbc.R800048200*
7. *Snell K, Duff DA. Alanine release by rat adipose tissue in vitro. Biochemical and Biophysical Research Communications. 1977;77(3):925-931. doi:10.1016/s0006-291x(77)80066-1.*
8. *Tischler ME, Goldberg AL. Leucine degradation and release of glutamine and alanine by adipose tissue. Journal of Biological Chemistry. 1980;255(17):8074-8081. doi:10.1016/s0021-9258(19)70609-7.*
